# Editorial: Monitoring, modeling, and mitigation in terrestrial ecosystems: microbial response to climate change

**DOI:** 10.3389/fmicb.2025.1717735

**Published:** 2025-10-22

**Authors:** Susheel Bhanu Busi

**Affiliations:** UK Centre for Ecology and Hydrology, Wallingford, United Kingdom

**Keywords:** climate change, microbial communities, soil microbiome, ecosystem processes, functional diversity

Climate change is driving profound alterations in terrestrial ecosystems, reshaping precipitation regimes, temperature patterns, soil chemistry and vegetation dynamics. Microorganisms, as central regulators of soil fertility, nutrient cycling and ecosystem stability, are both sensitive indicators of environmental change and active agents mediating ecosystem resilience. This Research Topic brings together diverse contributions that collectively highlight the ways in which microbial communities respond to and mediate climate pressures.

One strand of research emphasizes how innovative interventions can directly support soil microbial function under stress. The mini review by Davis et al. outlines the potential of biodegradable hydrogels, combined with microbial consortia, to counter soil dysbiosis. By enhancing water retention and providing microhabitats, such materials may stabilize microbial communities in degraded or drought-prone soils, offering a biotechnological route to climate-smart soil management. This focus on applied strategies signals a growing interest in microbial-based mitigation.

Complementing this, Morales-Fonseca et al. show how the functional diversity of soil macrofauna can help stabilize microbial communities during drought. Their experiments reveal that diverse macrofaunal assemblages help buffer microbial responses to stress, likely by mediating resource flows and habitat structure. This finding underscores the importance of considering whole soil biotic networks rather than microorganisms in isolation. It also links biodiversity at different trophic levels with ecosystem resilience under climatic stress.

Precipitation change, a key driver of microbial dynamics, features prominently in this Research Topic. Zhang N. et al. examine cbbL-bearing carbon-fixing microbial communities in an alpine wetland under simulated precipitation regimes. They demonstrate that rainfall variation not only influences microbial abundance but also regulates the functional potential of carbon fixation, with downstream consequences for carbon sequestration in high-altitude ecosystems. Similarly, elevational gradients provide natural laboratories for understanding climate impacts. Zhang B. et al. explore fungal community diversity at different soil depths in Tibetan forests, revealing how both altitude and soil depth jointly shape fungal assemblages. In a related study, Zhang Y. et al. focus on the northern slope of the Central Kunlun Mountains, showing that soil water availability significantly structures fungal communities along elevation. Together, these studies highlight the sensitivity of microbial communities to water dynamics across scales, from controlled experiments to broad landscape gradients.

The complexity of microbial responses is further illustrated by studies addressing multiple, interacting stressors. Xu et al. investigate how acidification and warming jointly affect denitrification and microbial community composition. Their findings reveal interactive effects on both microbial structure and the regulation of nitrous oxide fluxes, highlighting the challenges of extrapolating from single-stressor experiments to real-world ecosystems. The study exemplifies the need for experimental designs that incorporate climate complexity.

Another contribution explores microbial constraints within ecological engineering interventions. Liang et al. examine the decay of *Salix psammophila* sand barriers, widely used to combat desertification, and find that microbial communities experience metabolic limitations during decomposition. Such bottlenecks may slow nutrient cycling and undermine the long-term sustainability of barrier-based strategies. This study illustrates that even well-established land management practices require microbial considerations to ensure resilience.

## Toward integrated monitoring and mitigation frameworks

Taken together, these contributions illustrate three key advances in microbial climate research:

*1. Functional integration:* across studies, there is a shift from cataloging microbial diversity to linking community composition with functional traits (carbon fixation, denitrification and decomposition). This provides stronger predictive power for ecosystem processes.
*2. Cross-scale interactions:* microbial responses are shaped by interactions with macrofauna, vegetation, soil chemistry and hydrology. Understanding these linkages is critical for scaling from genes to ecosystems.
*3. Mitigation and management:* beyond documenting impacts, several contributions propose or test interventions—hydrogels, macrofaunal conservation and vegetation engineering—that point toward actionable strategies for sustaining soil health.

These themes align closely with the aims of this Research Topic: to monitor microbial responses to environmental change, model their ecosystem consequences and explore mitigation strategies. These advances also highlight areas for future progress. Multi-stressor studies will be essential to approximate the real complexity of global change. Longitudinal monitoring using functional markers can provide early-warning indicators of ecological tipping points. Linking microbial dynamics with biogeochemical models and Earth system frameworks will be crucial for incorporating microbial processes into climate projections. Finally, testing and refining mitigation strategies that leverage microbial ecology whether through biotechnology, biodiversity conservation, or engineered interventions will provide tools for managing soils under accelerating climate change.

Overall, this Research Topic underscores the central role of microorganisms in terrestrial ecosystem responses to climate change. The seven articles collectively demonstrate that monitoring microbial communities is indispensable for tracking ecosystem health, that functional modeling enhances predictive power and that microbial-informed interventions can support resilience. As climate pressures intensify, integrating microbial ecology into management frameworks and policy will be vital for sustaining soil health and ecosystem services into the future.

